# Editorial: Theoretical advances and practical applications of spiking neural networks, volume II

**DOI:** 10.3389/fnins.2026.1771268

**Published:** 2026-01-28

**Authors:** Gaetano Di Caterina, Malu Zhang, Jundong Liu

**Affiliations:** 1 Neuromorphic Sensor Signal Processing Lab, Centre for Image and Signal Processing, Electronic and Electrical Engineering Department, University of Strathclyde, Glasgow, United Kingdom; 2 Centre for Future Media, School of Computer Science and Engineering, University of Electronic Science and Technology of China, Chengdu, Sichuan, China; 3 Learning and Intelligent Systems Lab, School of Electrical Engineering and Computer Science, Ohio University, Athens, OH, United States

**Keywords:** artificial intelligence, brain-computer interface (BCI), encryption, event-based paradigm, neuromorphic engineering, speech enhancement, spiking neural network

## 1 Introduction

Spiking Neural Networks (SNNs) process information through discrete, time-dependent spikes, closely mimicking the dynamics of biological neurons. This temporal coding enables SNNs to capture rich spatio-temporal patterns and exploit event-driven sensing and computation, offering sophisticated information processing capabilities and practical efficiency at the same time.

The key strengths of SNNs reside in their biological plausibility and energy efficiency. SNNs can operate with significantly reduced power consumption compared to conventional neural networks and deep learning models. Advances in learning algorithms and theoretical and software frameworks have accelerated the development of SNNs, bringing neuroscience-inspired models to practical machine intelligence in engineering systems while also reducing the performance gap from deep neural networks (DNNs).

This Research Topic brings together cutting-edge research on a diverse set of practical applications of SNNs, such as an EEG-based brain-computer interface (BCI), data encryption, and speech enhancement, building on the theoretical foundations of SNNs and demonstrating their transformative potential in addressing complex challenges.

## 2 About the articles

The work by Abuhajar et al., entitled “*Three-stage hybrid spiking neural networks fine-tuning for speech enhancement*,” addresses the challenge of energy-efficient speech enhancement (SE) by introducing a three-stage hybrid training pipeline for SNNs. The proposed method first trains state-of-the-art Artificial Neural Network (ANN) models (namely Wave-U-Net and ConvTasNet), converts them into equivalent SNN architectures, and then fine-tunes them using a hybrid scheme, consisting of spiking-based forward passes combined with ANN-based backpropagation for gradient computation. Experiments on Noisy VCTK and TIMIT datasets show that fine-tuned SNNs significantly outperform baseline converted SNNs and achieve performance comparable to their ANN counterparts across metrics such as signal-to-noise ratio, STOI, and PESQ. Additionally, energy analysis reveals substantial efficiency gains, with fine-tuned SNNs consuming up to 7x less energy than ANNs.

In “*SpyKing—Privacy-preserving framework for Spiking Neural Networks*,” Nikfam et al. propose a novel approach to secure neural computation by integrating SNNs with Fully Homomorphic Encryption (FHE). Traditional DNNs face severe computational overhead in FHE, so this work investigates whether SNNs can offer better performance in encrypted environments by leveraging their event-driven paradigm. The authors implemented LeNet5 and its spiking counterpart, Spiking-LeNet5, and experiments were conducted on MNIST, FashionMNIST, and CIFAR10 datasets, systematically varying encryption parameters to balance security and efficiency. Results show that, while FHE dramatically increases computation time, SNNs outperform DNNs on encrypted data under certain conditions, achieving up to 35% higher accuracy for low plain text modulus values.

The article “*Spiking neural networks for EEG signal analysis using wavelet transform*” by Yuan et al. introduces the framework SpikeWavformer for BCI to overcome the reliance on manual EEG feature extraction and the high energy consumption of conventional deep learning models. SpikeWavformer uses a spiking wavelet self-attention mechanism that combines multiscale wavelet analysis with spiking attention to capture both global rhythmic patterns and local transient features in EEG signals. The authors evaluated the approach on the two benchmark datasets: DEAP for emotion recognition and KUL for auditory attention decoding. SpikeWavformer achieved state-of-the-art performance, with 76.5% accuracy for arousal and 77.1% for valence on DEAP and up to 98.6% decoding accuracy on KUL with longer decision windows. Additionally, the model demonstrated 7x greater energy efficiency compared to traditional ANNs.

In “*Multiscale fusion enhanced spiking neural network for invasive BCI neural signal decoding*,” Song et al. introduce the framework MFSNN to reduce cross-day variability in neural signals caused by electrode drift and neural plasticity and mitigate the high energy consumption of traditional ANNs. MFSNN integrates temporal convolutional networks, channel attention modules, and linear transformations within an SNN architecture. These components extract and fuse spatio-temporal features, while a spiking classifier enables event-driven, energy-efficient computation. Experiments demonstrate that MFSNN achieves robust performance, maintaining over 80% accuracy in cross-day decoding with minimal fine-tuning. Single-day decoding exceeded 95% accuracy, comparable to conventional models, while energy consumption was reduced by 90.9% compared to ANN-based counterparts.

## 3 Discussion and future trends

The articles clearly indicate that reduction in power consumption is the primary factor behind the use of SNNs. This also reflects the general interest in SNNs in an industrial context, where event-based sensing and processing are seen as key for effective AI. However, as Nikfam et al. reveal that encryption-based privacy-preserving computations introduce latency and computational overhead, this highlights the need for hardware acceleration. Indeed, this is an important point to raise, in that neuromorphic processing hardware is required to achieve the full potential of SNNs in terms of reduced computation and power consumption without compromising on task performance.

Another point to highlight is that, while the works presented in this Research Topic span across quite different application domains (such as EEE-based BCI, speech enhancement, and data encryption), they all aim to leverage two key strengths of SNNs, namely low-latency and biologically inspired computation. In these regards, the neuromorphic research community should investigate the definition of common design principles that can unify SNN architectures across the various application domains in order to help propel event-based sensing and processing beyond mere bespoke solutions for specific problems and application scenarios.

Adaptability and biologically inspired learning mechanisms can be key strengths for advancing SNNs. In fact, while in ANNs and DNNs adaptation needs to be specifically incorporated into the design of the architectures and the learning needs to be tailored to ensure tuning of weights and sections of the networks according to specific sequences of training steps, in SNNs the dynamics of spiking neurons and the built-in temporal understanding allow even simple feedforward SNNs to adapt to new input data at inference time. As the learning mechanism is somehow detached from the network architectures, this should prove to be an advantage for the neuromorphic research community and enable them to directly focus on the learning approaches.

## 4 Conclusion

SNNs are being used in a wider range of applications and, although there is still a scarcity of commercially available neuromorphic sensors, apart from event-based cameras, the research community has put forward various other methods to generate event data, so that SNNs can be used effectively with conventional, i.e., non-spiking, data. Nonetheless, it is still hoped that novel truly neuromorphic sensors will be developed in the future to go beyond the limitation of current sensing modalities and let the event-based processing paradigm fully flourish. Moreover, there are still numerous promising research directions to explore, including hardware acceleration for neuromorphic processing, algorithmic innovations, and hybrid ANN-SNN frameworks, which will allow the effective deployment of end-to-end neuromorphic pipelines in real application scenarios.

